# The plant body as a network of semi-autonomous agents: a review

**DOI:** 10.1098/rstb.2018.0371

**Published:** 2019-04-22

**Authors:** Beata Oborny

**Affiliations:** 1 Institute of Biology, Loránd Eötvös University, Budapest, Hungary; 2 GINOP Sustainable Ecosystems Group, Centre for Ecological Research, Hungarian Academy of Sciences, Tihany, Hungary

**Keywords:** agent-based model, dynamic network, plant development, clonal plant, phenotypic plasticity, adaptation to heterogeneity

## Abstract

Plants can solve amazingly difficult tasks while adjusting their growth and development to the environment. They can explore and exploit several resources simultaneously, even when the distributions of these vary in space and time. The systematic study of plant behaviour goes back to Darwin's book *The power of movement in plants*. Current research has highlighted that modularity is a key to understanding plant behaviour, as the production, functional specialization and death of modules enable the plant to adjust its movement to the environment. The adjustment is assisted by a flow of information and resources among the modules. Experiments have yielded many results about these processes in various plant species. Theoretical research, however, has lagged behind the empirical studies, possibly owing to the lack of a proper modelling framework that could encompass the high number of components and interactions. In this paper, I propose such a framework on the basis of network theory, viewing the plant as a group of connected, semi-autonomous agents. I review some characteristic plant responses to the environment through changing the states of agents and/or links. I also point out some unexplored areas, in which a dialogue between plant science and network theory could be mutually inspiring.

This article is part of the theme issue ‘Liquid brains, solid brains: How distributed cognitive architectures process information’.

## 1. Introduction

The movement of plants, in particular their responses to the environment by growth, has fascinated natural scientists for a long time. Charles Darwin wrote the following in *The power of movement in plants* (1880, co-authored with his son, Francis; [1]),

The habit of moving at certain periods is inherited both by plants and animals; and several other points of similitude have been specified. But the most striking resemblance is the localisation of their sensitiveness, and the transmission of an influence from the excited part to another which consequently moves. Yet plants do not of course possess nerves or a central nervous system; and we may infer that with animals such structures serve only for the more perfect transmission of impressions, and for the more complete intercommunication of the several parts.

Since the birth of these sentences, many botanists and plant ecologists have made experiments on plants' perception of and responses to the environment from the whole-plant scale, through organs, tissues and cells, to the finest details of molecular mechanisms. These investigations confirm Darwin's idea, and show that the ‘transmission of influences’ has sophisticated mechanisms within the plant body. The subject of the present paper is a macroscopic view of the plant, focusing on the highest organizational levels: the whole plant, subdivided into developmental modules (see their definition below). I propose that network theory provides a convenient tool for studying the ‘intercommunication of the several parts’. Accordingly, the modules will be represented as nodes, and the interconnections as links in a network.

These few pages cannot encompass the whole variety of plant growth forms, and all the interactions between the plant and the environment. The review is restricted to some remarkable examples in terrestrial vascular plants. The main focus is on foraging behaviour, by which the plant explores and exploits resources in the habitat [2,3]. The root system typically searches for water and mineral nutrients, while the shoot system forages for light. The environment usually provides various cues about the locations of these resources within the sites that have already been occupied, and also in the neighbourhood that can potentially be reached by further growth.

It is a challenging task to describe the environment from the plant's point of view, i.e. to identify the relevant cues, and to study the spatial and temporal scales of perception and response in each species [4]. In the past few decades, many experiments have been conducted to examine plants' reactions to environmental heterogeneity. The studies have clearly demonstrated that plants are generally able to gather information from the environment actively, and many species can adjust growth not only to the currently available but also to the anticipated resources [5–12]. A conspicuous example for active information acquisition is the exploratory growth of stolons, whereby the tip of each stolon can move or stop according to the local conditions, and can send back signals to the rest of the plant [3]. The plant processes the acquired information through various correlative interactions between its parts [6,10,11,13,14]. Epigenetic inheritance permits the storage of information about past states, and the transmission to newly developing parts [14,15]. In general, the plant's growth form is not a passive ‘mirror image’ of the external world: each plant individual interacts with its environment at multiple points actively, in a coordinated manner.

In this paper, I review some typical challenges posed by the environment, and discuss the plant's potential and limitations in solving these tasks (see more detailed reviews in [7,16]). I re-consider the plant's modules as a group of cooperating agents, which functions through distributed control. Accordingly, I describe the plant as a network of agents and mention some examples of questions that could typically be answered by network modelling. Finally, I discuss some general issues (e.g. cooperation versus competition between the agents) that are applicable to other, similar systems as well (e.g. to ant colonies).

## 2. Modularity and adaptation

Multicellular organisms can be divided into two major groups from the aspect of development: unitary versus modular ones. Mammals, arthropods and molluscs are typical examples of unitary organisms. Their ontogeny, starting from a single cell, realizes a definite body plan, in which the number of organs is strictly defined. By contrast, plants are modular organisms (together with corals, sponges and some other animal taxa). Their ontogeny progresses by the re-iteration of finite developmental programs, each producing a module [17–19]. The structure within each module is well-defined, but the system of modules can be flexible, similarly to a construction toy.

The production of modules is typically repeated throughout the lifespan of the organism (i.e. modular organisms have open developmental programs; for more details see [19,20]). The growth of the whole body can be described by the birth and death of the modules (i.e. module demography, see [21,22]). Birth can lead to branching. The positions of new modules are determined by architectural rules, which include the probability and angle of branching [23–25]. On this basis, several researchers have described the modular development of plants by generative algorithms [3,26,27]. One of the pioneers in this field was Aristid Lindenmayer, who invented a formal language as a theoretical basis of algorithmizing plant growth (see the book *The algorithmic beauty of plants* [28]). Nowadays computer programs can simulate the development of some species with life-like accuracy (e.g. *Trifolium repens* in [29]).

**Figure 1.**
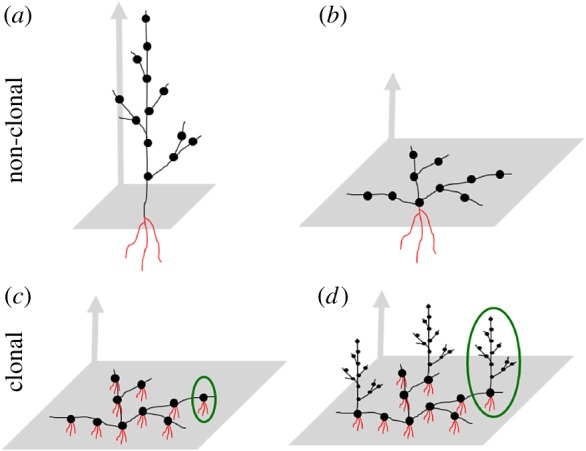
Some typical growth forms in plants. Each metamer consists of a nodus (black dot) and an internodium (black line). Roots are drawn in red. The horizontal plane at the ground level and the vertical direction are indicated in grey. The upper row shows non-clonal growth forms: (*a*) erect, and (*b*) prostrate. The lower row presents clonal plants in which the ramets are formed from (*c*) metamers or (*d*) branches consisting of multiple metamers. An example of a ramet is outlined in green in (*c*) and (*d*).

The present paper applies a simpler representation of the plant body, in order to search for common features in various species. Most of the examples will be taken from the shoot systems of vascular plants, in which the modular structure is well recognizable. In general, the basic ‘building block’ in the shoot system is the metamer [30]. It consists of a nodus,^1^ an internodium, and those organs that are attached to the nodus (leaves, spines, etc.). Typically, each nodus contains at least one branching point (lateral bud). The potential for branching at a particular point may or may not be used by the plant, depending on its internal state and the environmental conditions [31]. Figure 1*a* shows a simple example for a branching structure. Not only the shoots but also the roots form branching structures, although the boundaries between the growth units are less visible (see [32] about modularity in roots). Modular development implies that not only the quantity but also the qualities of the modules can change over time. They mature and can specialize for various functions (reproduction, storage, etc.) over time.

From an ecological and evolutionary perspective, one of the most important characteristics of modular organisms is their open developmental program. The modules are continuously produced and die; meanwhile, each module is interacting with the environment. Thus, morphogenesis and selection progress in interaction, on the move. Successful directions of growth can be maintained while unsuccessful ones can be abandoned [11,33]. More organs of resource uptake (e.g. leaves) can be accumulated in those places that are richer in the resource (e.g. light; [2]). Altogether, the individual can adjust its development to the environment continuously, even when the environment is changing. Beyond individual adjustment, evolutionary adaptation is also a key topic of research. The aforementioned developmental rules, viz. (1) those that concern the modules' demography (birth and death), and (2) the architectural rules, which govern the placement of newly produced modules, are heritable, and are subject to natural selection. Therefore, the sets of rules can change via Darwinian evolution [3,11,16,19,23,34,35].

Both kinds of rules can be environment-dependent. For example, in a canopy gap with relatively higher light (1) the probability of module birth may be higher and/or (2) the stolon length may be shorter than under the closed canopy (e.g. *Brachypodium pinnatum* and *Agrostis stolonifera*, respectively [36]). In a model, this can be represented as a conditional (if–then) rule. In biology, such phenotypically plastic reactions to the environment are customarily represented by a function, the reaction norm [37]. Reaction norms are subject to evolution.^2^ Altogether, it is quite important to distinguish between two kinds of processes: adjustment and adaptation. Adjustment to the environment is a process within the individual, enabled by the environment-dependent rules. Adaptation is an evolutionary process, involving multiple generations of individuals, and can involve environment-dependent or independent rules. An example of adaptation by environment-independent rules is the optimization of space-filling by rigid branching patterns (see, for example, the regular hexagonal branching pattern in the rhizome system of *Alpinia speciosa*; [23]). Most plant species show a mixture of rigidity and plasticity (see a general review about the adaptive value of mixing rigidity with plasticity in [38]).

## 3. Module autonomy and clonal plants

Modules are produced by the repeated activity of a set of totipotent cells, located in meristems. When a new module is produced, it is necessarily subsidized by the parent module, and often by more, older ones [39]. It is interesting to study the pattern of physiological connectedness between the modules. Vascular plants are very diverse in this regard [40–42]. The main division line is between clonal and non-clonal plants (figure 1). In non-clonal plants, physiological connections persist throughout the modules' lives; while in clonal plants, some parts of the body are able to attain complete physiological autonomy over time. The autonomous parts, by definition, possess all the organs that are characteristic for the actual species (including roots in the case of terrestrial plants). By these means, the plant reproduces vegetatively [18,34].

The study of clonal organisms directed the attention of researchers towards the nature of individuality [20,34,43]. In clonal species, the individual in the genetic sense (the genet) is not the same as the individual in the physiological sense (the ramet). The former is defined as the product of a single zygote (illustrated by the whole plants in figure 1*a*–*d*). Ramets are those subunits within the genet that can become physiologically autonomous (outlined in green in figure 1*c*,*d*). Plants show an amazing diversity in the levels of hierarchy contained in a ramet. In some species, for example, in *Trifolium repens*, it is the metamer, which can become autonomous (figure 1*c*). In others, it is the branch or branching system (e.g. in *Aster lanceolatus*; figure 1*d*). The common feature is fragmentation of the genetic individual into multiple physiological individuals, and it is achieved in diverse ways.^3^ Owing to this diversity, I use the term ‘module’ in a broad sense: any subunit within the hierarchy can be a ‘module’. Ramets are those naturally occurring minimal modules that are potentially autonomous in a fully developed (non-juvenile) state. This potential can be tested, for example, by cutting the connections [39,45,46].

**Figure 2.**
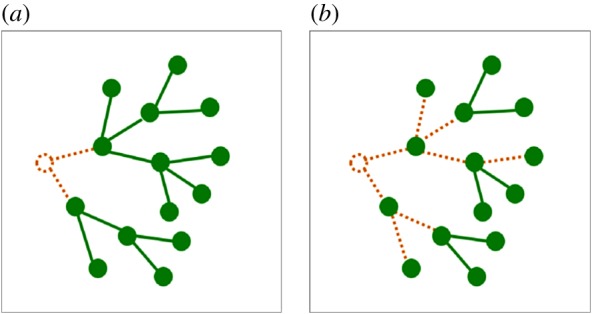
A snapshot from the growth of a clonal plant (a view from above). The ramets are denoted by circles, and their links (e.g. stolons or rhizomes) by lines. The living/dead parts are solid/dotted lines in green/brown colour. The clone changes by the birth and death processes of the ramets and links. The birth of a ramet necessarily co-occurs with the birth of a link. The death of a ramet causes the death of its links (see the brown ramets). But the death rate of links (*d*), relative to the death rate of ramets, is highly variable among the species: (*a*) an integrator (low *d*), and (*b*) a splitter (high *d*). The integrator consists of larger fragments than the splitter; therefore, it can perceive its environment in a more coarse-grained manner than the splitter (see more about the grain of perception in [4,7,16,50–52]).

In some species, the potential is not manifested in normal conditions, and becomes important only when the connection is severed (for example, by a herbivore). These species are often called ‘integrators’. In others, fragmentation occurs frequently, and may even be developmentally programmed [47]. These species are ‘splitters’ [48,49]. Whether a species is considered to be an integrator or a splitter depends on the longevity of the links relative to that of the ramets (figure 2; [39,51]).

Integration versus splitting primarily depends on the species, but can be influenced by the environment as well. For example, genets of *Fragaria chiloensis* growing in a grassland split more than genets in a woodland [53]. This indicates the capability of adaptation to the habitat by changing the degree of integration (see also [16,25,48,51,54–58]). In summary, it is worth considering at least three hierarchical levels in a clonal plant: the genet, the fragment and the ramet levels. The ramets are non-autonomous at the start of development. Later they can become fully autonomous or remain semi-autonomous. Full autonomy is more characteristic in splitter species, but may occur in integrators as well in the case of injury (e.g. [44]). Semi-autonomy means that the ramet receives and/or sends some material from/to other ramets, but can also take up some of the resources independently of the others.

Figure 1 illustrates the relationship between the two major domains, the shoot and the root system, in non-clonal versus clonal species. This spatial relationship is crucial, because the shoot versus root system take up different resources, and need to exchange them (cf. [13,59]). Non-clonal plants have a single connection between the shoot and root system. In clonal plants, each ramet can possess a shoot and a root system. Therefore, the genet is largely freed from the size constraints of non-clonal plants [19]. Clones with amazing sizes and ages have been discovered in various species. For example, a single genet of *Populus tremuloides* was found to cover approximately 81 hectares, and was estimated to be more than 10 000 years old. A genet of *Gaylussacia brachycera* was 1980 m in diameter, and its age was estimated at 13 000 years [60]. Genets that are several hundred years old and consist of hundreds of ramets have frequently been observed in various species [61]. Obviously, the genet's lifespan can be much longer than the ramets'. During the birth and death processes, the genet can move over considerable distances, and meet various environmental conditions. Several studies have demonstrated a considerable variation in the amount of resources above and below ground even on fine (centimetre or decimetre) scales in grassland and woodland habitats (see a review in [59]). It is an exciting subject of research how the plant copes with this environmental variability by adjusting its growth to the spatial and temporal pattern of resources. I believe that a common theoretical framework, which is applicable for a broad variety of plants, would facilitate this research. I think that network theory is a good candidate for a common ground.

## 4. A network-based approach

I suggest representing the plant's body as a network of interacting agents. The agents are those parts of the plant that take up resources and/or information from the environment. The links between the agents represent the potential pathways of the exchange of resources and information.

The idea of a functional subdivision of the plant body, according to the strength of interaction with the environment, is not new in the literature of plant ecology. Adrian D. Bell proposed this kind of partitioning in the 1980s [27]. He primarily focused on the plant's resource acquisition, and distinguished between two main functional constituents, ‘feeding sites’ and ‘spacers’. As he described, the function of a feeding site is to take up resources from the environment, while the task of spacers is to place the feeding sites into locations that are relatively rich in resources, and to serve as channels for the flow of resources (see also [62,63]). The idea of studying the flow of information appeared even earlier, in the works of Darwin, who discussed ‘*the transmission of an influence’* from one part of a plant to another (for example see [1, p. 572]). More recent examples about the importance of information flow include the correlation of development between the parts of the plant by means of hormones [6,10,40,62], and the transfer of warning signals in the case of herbivore attack [63–65]. The idea of considering the clonal plants' stolon systems as communication networks has been proposed in the context of warning signals [63–65]. In these studies, the main focus was on the importance of links, and less emphasis has been given to the states of the nodes in the network. The objective of the present review is to unite the previous approaches, and complement them. Accordingly, (1) both the nodes and the links are considered, (2) both the resources and information (specifically, about the distribution of the resources) are taken into account, and (3) the network structure is related to the environment's spatial structure. (4) Since the agents and links are produced and die continuously, the network dynamics are also studied.

Some clonal plant species provide excellent examples for the study of such networks. In a simplified view, the ramets are the nodes (the agents), and the connections between the ramets (stolons, rhizomes, etc.) are represented as links in the network (figure 1*c*,*d*).^4^ It is essential to consider that the ramets may be in different local environments (e.g. at high versus low resource level). To represent this, the network can be embedded into space. Spatial embedding makes it possible to consider that the resource distribution may be spatially autocorrelated.

## 5. Tasks and sources of information

Living organisms, in general, have numerous tasks in their natural habitats. Some are related to the resources that are essential for life: (1) to explore the resources, (2) to exploit those resources that have been found, and (3) to defend them against competitors. In addition, there are many tasks that do not directly increase the uptake of resources, but necessarily consume resources: for example, sexual reproduction, and defence against parasites and herbivores. The examples I present below are primarily related to (1) and (2). These activities are linked in the literature of behavioural ecology under the keyword ‘foraging’ [3,24,62,66].

The adaptive value of any of these activities is measured by their impact on fitness in the actual environment. In clonal organisms, the genetic individual's (the genet's) fitness should be maximized, which can be achieved even by sacrificing some of the ramets (cf. [18]). The ramets, as semi-autonomous or autonomous agents, take up resources from the environment locally. In the case of semi-autonomy, they can redistribute the resources among themselves, and use them for developing further ramets. This is a sophisticated dynamic optimization problem: the question is how to invest the current amount of resource, at any point of time, in order to maximize the gain in the end [16,47,56]. The time horizon is quite important: computer simulations have demonstrated that a plant growth form which is advantageous for a small number of ramet generations may be disadvantageous later [67] (see also an experiment: [68]). The individual's endpoint is the death of the genet, but the initiation of new genets by sexual reproduction should also be taken into consideration (see [69,70] about modelling reproductive allocation in clonal plants).

Foraging in (unitary) animals is usually achieved by moving across the habitat. In its simplest model, the animal can choose between two activities at any point of time: (1) to move, and thus explore a new resource patch, or (2) to stay and exploit the current patch. In plants, these activities correspond to allocating resources (1) into the growth of a spacer (a link), versus (2) into a feeding site (an agent) [3]. Plant foraging is particularly interesting because the animal can move *or* stay, while the plant can move *and* stay at the same time, leaving one agent at the original location, and developing a new one at another site (figure 2). As a plant physiologist put it, ‘plants have multiple mouths’ [40]. More generally, each agent in the network can be considered as a sampling point from the environment, which can collect local information and resources.

The most relevant information for the plant, in the context of foraging, is about the distribution of resources. Some resources can be perceived via specific cues, which are not the same as the resource itself. For example, parasitic plants can sense the direction of the host plant via chemical cues [71]. Gaps in the canopy can be sensed by a change in the spectral composition of light (higher red/far-red (R : FR) ratio; [2,6]). The plant can even be ‘deceived’. In some experiments, the photosynthetically active radiation (the resource) was decreased, while the R : FR ratio (the cue) was increased artificially. The plant responded as if it had more resource [8,72]. The specific cues may even allow anticipation. For example, it has been demonstrated that *Portulaca oleracea* can sense an increase of R : FR ratio in the light reflected from a surface on one side, and responds to it by growing toward the stimulus. Such anticipatory cues enable the plant to avoid competitors before competition occurs [11].

**Figure 3.**
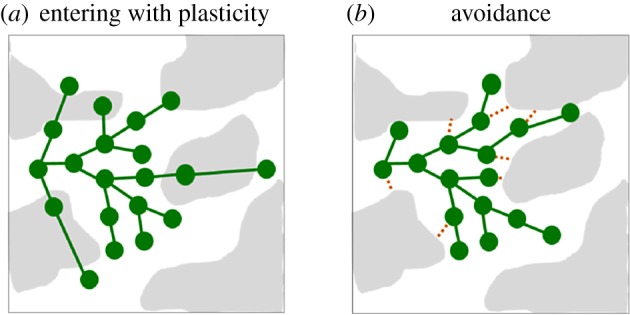
Examples of foraging strategies in heterogeneous environments. The notations are the same as in figure 2. Favourable/unfavourable sites are white/grey. The figure illustrates two dichotomies: avoiding versus entering into unfavourable patches, and plastic versus rigid growth of the surviving parts. Various combinations are possible; these are only examples. In (*a*) the plant can enter into unfavourable patches by plastic growth, while in (*b*) it avoids the unfavourable portion of the environment by getting rid of those parts that would grow into unfavourable terrain. In (*a*), phenotypic plasticity concerns the architecture (by modifying the stolon length) and the birth rate of ramets. In (*b*), plasticity is manifested in the death rates of stolons and ramets.

In other cases, the cue is simply a change in the amount of resource. For example, a decrease in the amount of soil nutrients reduces the probability of branching in *Glechoma hederacea*, and also induces the elongation of stolons [62]. Therefore, the genet can grow through the unfavourable patches, placing relatively more ramets into the favourable ones (see a general illustration, not of this particular species, in figure 3*a*). Computer simulations have confirmed that this behaviour is adaptively advantageous in almost any kind of patchy habitat [4]. It is particularly interesting in the example of *G. hederacea* that the availability of a below-ground resource could induce a specific morphological change above-ground, in the shoot system. Information transfer in the opposite direction has also been observed: the quality of light above-ground influenced the development of roots in *Festuca rubra* [73]. This underlines the importance of information processing within the ramet (the agent).

Similar kinds of local (agent-level) responses have been studied by numerous experiments (for reviews see [2,36,37]). Fewer experiments have investigated larger groups of agents (for example [45,68,74–76]), although various emergent properties have been observed in natural habitats. For example the collective behaviour of the agents can produce characteristic broad-scale spatial structures [16,25,48,77], including ‘fairy rings’ [78–80]. The stay-and-move nature of space occupation enables the plant to compare the environment at different locations, for example, to sense gradients [5,11,81,82]. Accordingly, the group can find and move toward the relatively more favourable direction [81,83], performing a ‘hill climbing’ on the favourability landscape. This kind of hill climbing is possible even in the lack of sensing the gradient directly. The plant's method is similar to the algorithm in many Artificial Intelligence models: some random variation is generated locally; the unsuccessful agents are discarded, while the successful ones are kept.

Some studies have suggested modelling the growing tips of roots as a swarm [32,84,85]. This idea expresses clearly the importance of the collective behaviour of interacting agents within the plant. In agreement with this, I propose to include the shoot system in the considerations as well. The shoot tips have sophisticated kinds of searching behaviour. In addition, I suggest considering the other modules as well as parts of the swarm: not only the tips but also the older parts of the root and shoot systems contribute actively to the search for favourable sites. Before detailing this contribution, let me briefly describe the challenges posed by the environment to the foraging plant.

Most habitats are patchy in terms of the availability of resources. Below ground, significant heterogeneity in the availability of water and nutrients (nitrogen, phosphorus, etc.) has been detected even on the spatial scale of single ramets [86]. Above the ground the availability of light can be similarly patchy, owing to the presence/absence of competitors [2,5,7,11,87,88]. For clonal plants, which grow horizontally to a considerable extent, the growth process is similar to path-finding in a labyrinth of favourable/unfavourable sites [33]. This is particularly important in those species that tend to avoid the unfavourable sites (figure 2*b*). An additional difficulty is that the environment can change owing to competitors and disturbances; consequently, the plant has to find ways in a labyrinth in which new pathways are opening and old ones are closing over time. ‘Ways’ refers to the fact that each branch has to find its own route.

To solve this task, the plant has a limited amount of resource at a time, which can be allocated into the growth of different structures. Let me collect some characteristic dilemmas posed by the environment to the growing plant. (1) The basic dilemma of foraging is whether to stay or move (see above). (2) When moving, it is a matter of decision whether to avoid unfavourable patches by stopping at those patch boundaries where the plant would move from favourable to unfavourable conditions (see the text above about the plant's ability to compare sites). The alternative is to enter into the unfavourable patch, trying to reach the next favourable one by growth [89]. (3) An almost ever-present possibility is to jump out of the present region by seed dispersal. In this manner, a new genet is established, usually in an uncontrolled direction, and at a larger distance compared with clonal growth.

These choices are represented by norms of reaction (see above), which describe how the plant responds to various local environments by phenotypic plasticity. The norms can evolve; thus, the plant can adapt to the environment, in particular, to the spatial and temporal pattern of resources. A related question is how reliable the environmental cues are. A developmental decision, made at a particular point in space and time, has consequences on the plant's form and function further away and later. For example, there is a considerable time lag between the initiation of a stolon and ending its growth by rooting and developing a new ramet [90]. The success of phenotypic plasticity, in general, hinges on the reliability of the inducing signal, with respect to the future selection [4,91,92]. To study this relationship explicitly, I proposed a measure of the predictability of the environment on the scale of plant growth, i.e. from the organism's perspective. It expresses the information content of a local signal relative to the global pattern of the resource availability in space and time [4]. (See also a general model about biological organisms as ‘guessers’ in [93].)

The local responses, on the level of links and/or agents, have emergent consequences on the level of the network, leading to typical behavioural types. Some contrasting types have been described on the basis of observations in natural habitats and experiments in planting pots. Some examples for these network-level alternatives are: (1) the genet can be fast and imprecise versus slow and precise in foraging [59]; (2) occupy a relatively small area by tightly packed agents versus a larger area by loose packing; (3) defend the occupied area from competitors versus letting them in, occupying new territories meanwhile [11,94]. The combination of (2) and (3) is often mentioned as the phalanx versus guerrilla dichotomy in the classification of plant growth forms (cf. [22,76,95–97]). The existence of these behavioural types is a key to understanding the self-organization of plant communities. Species coexistence strongly depends on the spatial matching of growth forms [16,25,76,94,98,99]. For example, one of the species may fill the gaps in the canopy of the other [97,100]. The study of these ‘matching’ mechanisms is crucial for understanding the diversity and stability of ecosystems [16].

## 6. Potentials and limitations in the plant's behaviour

The systematic study of plant behaviour has been proposed and initiated in diverse ways by many authors in the past three decades [5,11,19,24,37,62,75,101]. Even ‘plant intelligence’ has emerged as a keyword in the literature [102,103]. I think that this expression is not an exaggeration, comparing plants with some similar systems in Artificial Intelligence research. Plants can display complex kinds of behaviour, and adjust them to the environment. Some authors have classified them as non-cognitive behaviour, in the sense that there is no central information processing and storage [101]. Other authors have extended the definition of cognition, on the basis of information theory, to include agents that are capable of sensing their environments and reacting to changes in highly adaptable ways [93]. According to the broader definition, plants can be considered as groups of cognitive agents. It is important to note that the lack of central control, i.e. the distributed nature of the plant's functioning, is not a weakness, but has considerable advantages.

Modular construction of the plant body enables some special ways of interaction with the environment:

(A) Each module can be considered as an individual point of interaction. The locations of these points can be actively influenced by the plant through its environment-dependent (i.e. plastic) rules of growth (e.g. figure 3).
(B) According to the environment at these locations, the same genotype can be expressed in various phenotypes simultaneously. For example, a genet can contain vegetative and reproductive ramets [31].
(C) The modules can share resources and information through their connections.

A and B imply that each module can be considered as an individual ‘guesser’ (*sensu* [93]), and C enriches the set of opportunities by adding that the ‘guesses’ can be shared. Furthermore, a combination of B and C can result in a division of labour between ramets [9,104,105]. For example, in *Trifolium repens*, *Fragaria chiloensis* and other species, experiments have shown that each ramet specialized for the uptake of the locally more abundant resource, and they exchanged their resources through the connections [46,104]. Computer simulations have shown that relatively simple rules in B and C can enhance the genet's performance significantly in a broad variety of habitats [106].

Foraging for resources is very efficient in some clonal plant species. The efficiency is usually characterized by the ratio of ramets placed into favourable versus unfavourable sites. For example, Roiloa & Retuerto [68] conducted an experiment in which a growing *Fragaria vesca* genet was surrounded by six patches of soil with equal sizes and various nutrient contents. Initially, the plant strongly preferred the most nutrient-rich patch, placing the majority of its ramets there. When the best one had been densely occupied, and even the second best contained lots of ramets, the plant started to grow into the worse kinds of patches. In general, in spite of the rooted nature of ramets, the genet is remarkably mobile in some species, and is capable of active habitat selection (see a review in [3]), provided that the habitat patches are on the scale of its perception and response [4]. For example, Wijesinghe & Hutchings made a series of experiments in which the same amount of resource (nutrients in the soil) was distributed in various spatial patterns [52]. Changing the grain of habitat patchiness caused more than a twofold difference in the produced biomass. That is, the plant's resource utilization was significantly higher when the resource was distributed in a spatial resolution that matched the plant's ability of perception and response. The response can be particularly limited in ‘labyrinth’ situations (figure 3*b*). Simulations have demonstrated that the architecture of the plant can seriously limit its ability to use the total amount of resource [33]. For example, it cannot make sharp turns and thus find some pathways.

The need for a proper spatial and temporal scale in the environment's heterogeneity is a serious limiting factor for the plant [4,7,16,107]. The relevant resolution depends on the size of the ramets (see B above) and on the length of connections (see A). In addition, C can significantly rewrite the external pattern of resources internally, within the plant body. For example, water can move across more than 80 cm within the stolons of *Lycopodium annotinum* [108]. Physiological integration within larger fragments causes a coarser-grained perception and response to the environment (cf. figure 2).

Naturally, foraging is not the only optimization task even within the context of resource economy. The resources taken up from the environment at any time should be optimally distributed among three major tasks: (1) the production of new modules, (2) maintenance of the existing ones, and (3) repair. The functional robustness of the network, even in the case of environmental adversities (draught, herbivore attacks, etc.) is a key to the genotype's fitness.

In general, plant behaviour is based on growth and developmental responses to the environment [5,19,37,101,102]. It is an attractive feature of plants that, owing to the move-and-stay nature of responses (instead of move-or-stay), not only the actual behaviour, but also a part of the past behaviour can be seen. Colleen K. Kelly expressed this by writing about the plant's past foraging decisions ‘etched’ into the present form [109]. I would add that mostly the successful trials can be seen. For example, a tree's present branching structure reflects its past decisions about branching, but most of the unsuccessful branches have disappeared by now. The complexity of plant behaviour originates from the fact that many events can happen simultaneously.

## 7. Perspectives in the network view

What do we gain by viewing the plant as a network of agents? I think this is not just a metaphor, but has practical consequences. The rich methodology of network theory could facilitate the study of plants for the following reasons.

(1) The individuals (genets) may consist of so many modules that it is difficult to overview the whole system's structure.
(2) The transmission of signals and the flow of resources between the modules makes it important to investigate how an event happening at one module influences the other, directly or indirectly connected modules.
(3) There is a strong demand in ecology to classify species according to their functionally relevant traits [88,99,110–112]. Many network properties are functionally important in plants (see more about this below). On the other hand, experiments can usually handle only relatively small pieces of the network (e.g. pairs of ramets, or small groups of connected ramets in clonal plants; see a review in [16]), and the observations are typically much shorter than the lifespan of the genet. Network theory may help to extrapolate for larger system sizes and longer times. Thus it can help to identify the functionally relevant traits.
(4) Representing the plant's body as a network can provide a common ground for comparing (a) different species, (b) different genotypes within the same species, or (c) different states of genets or fragments, for example, according to their age, nutrient supply or environmental stress.

The plant's body, as a network, is essentially a tree in the sense of graph theory, i.e. it does not contain any cycle (unless we add fungal mycelia to the potential links; e.g. [113]). This may seem to be a simple kind of network. This is not the case, however, if we consider those diverse phenomena that unfold on the basis of this tree structure.

(1) The network is dynamically changing owing to the birth and death of ramets and links. Meanwhile, it is likely to get fragmented, i.e. to fall apart into independent sub-graphs (figure 2). This is a ‘forest’ structure in graph-theoretical (but not in botanical) terms.
(2) The nodes can be in various states. For example, they can specialize for sexual reproduction or for the uptake of a resource (see §6 about the division of labour).
(3) The links can also vary, e.g. in their transmission capacity. Typically those pathways get stronger which lead to relatively fast growing, and thus, more promising branches [41].
(4) The above-mentioned states of the nodes and links can depend on the age or nutritional state.
(5) Accordingly, the source–sink relations of resources are dynamically changing [82].
(6) In many species, the strength of the acropetal versus basipetal transport differs [7,82]; therefore, the graph is directed.
(7) The three kinds of responses to the environment listed in the previous section (A–C) imply that the network is capable of integrating information on multiple scales.
(8) The network is embedded in space. The interaction between the plant's structure and the habitat's structure is an exciting topic of research [16]. Not only can the environment affect the plant, but also the plant can affect the environment. One of the non-local, network-level effects is that the plant can move a significant amount of resource from one site to another through its vascular system. Therefore, it can actively increase or decrease the suitability of the habitat in certain sites. A characteristic example for the increase of suitability is when a clonal plant is gradually invading into a rock surface, and gradually ameliorates the living conditions over the surface (see a collection of similar examples in [100]). An example of the decrease of suitability is the depletion of soil resources in the interior of a fairy ring [80].

These examples indicate the need for a firm theoretical foundation for the study of plant modularity. I think that network theory could valuably contribute to this foundation. I mention some examples below of network-related questions about clonal plants. As the clonal plant is hierarchically organized (ramet–fragment–genet; figure 2), I have divided these questions into two groups: (1) characterizing a fragment by its constituent ramets, and (2) characterizing a genet by its fragments. In both cases, the question is about a variable's statistical distribution within the group.

(1) *From the ramets to the fragment*. One of the basic local (i.e. ramet level) properties is the number of attached links, i.e. the degree. The degree-distribution within the fragment could be used, for example, for characterizing its linearity. A related observable is the perimeter to core ratio. Let me define the perimeter, in the present context, as the set of ramets with degree 1. The perimeter to core ratio is particularly important when the two kinds of ramets can have specific functions or spatial positions. For example, the ramets in the perimeter may be less likely to have flowers, or may be spatially more peripheral, which increases the chance of having more neighbours that belong to different species in the plant community. It would also be interesting to study the graph distances between the ramets, because the flow of material or the spreading of pathogens within the plant is generally distance-dependent. The graph distance between ramets *i* and *j* is defined as the number of links in the shortest path connecting *i* and *j*. (Note that graph distance and spatial distance are different concepts.) A fragment can be characterized by its graph diameter, i.e. the maximum graph distance that occurs between any two ramets. Bigger diameter means that more ramets can influence each other's state sequentially (e.g. by resource transport or infection by a pathogen). The importance of each ramet in the network could be characterized, for example, by its betweenness centrality. This is defined as the number of shortest paths that go across the ramet. It would be interesting to study whether this kind of importance correlates with any other attribute of the ramet, for example, its size. Such correlations may cause characteristic degree distributions (for example, larger ramets maintaining more connections leads to preferential detachment). The death of links (stolons, rhizomes, etc.) has been a subject of many studies (see [39] for a review), but, as far as I know, the preferential versus non-preferential nature of detachment has not been studied in plants. I think this would be an exciting new direction of research.
(2) *From the fragments to the genet*. Fragments originate from the death of links. Their statistical properties also depend on the birth and death of agents (cf. figure 2). One of the significant genet-level properties is the size distribution of the fragments. As the links transfer resources and information, it could also be fruitful to reveal the pathway's structure. Large fragments may even be modelled as percolation networks (on tree structures). It is interesting to ask how far a resource, taken up at a point, can get within the network. Both the width and the depth of the network are biologically relevant. For example, embedding the network into space, the graph's diameter sets an upper limit to the distance within which resources can be translocated, and information can spread. In more complex network models of the plant body, it may also be considered that the flow of material and information in the basipetal versus acropetal direction can be different [7,82]. Such models would be based on directed, weighted graphs. Convergence and divergence (in the sense used in neural networks) may characterize the plant in terms of basipetal and acropetal flow, respectively. Since the maintenance of the links and the transport itself can be costly, the optimal strategy of the plant in a habitat is strongly influenced by these network-level properties.

A thorough understanding of the network structure in clonal plant species may facilitate ecological fieldwork as well. It is not easy to observe the links in some species, especially in rhizomatous ones. Assuming that the sampling capacity is limited, it is important to ask how to collect samples from a network, in order to estimate a trait in the rhizome system with acceptable accuracy. How robust is a trait to missing some links? The topics mentioned in this section are only examples from the potential fields of research at the interface between plant science and network theory. These fields are largely unexplored. I believe that crossing the disciplinary boundary could lead to exciting new discoveries.

## 8. Outlook

The plant body is one of the systems that can solve complex tasks without any central control. This capability is common in many biological and artificial systems (see for example [114] in Artificial Intelligence research). The most similar among the biological ones is, perhaps, the collective behaviour of colony-forming organisms, like ants [115,116] or colony-forming slime moulds [117]. I believe that the involvement of plants in the research of swarm intelligence would be mutually inspiring for both fields of research. The objective of the presently proposed agent-based approach is a step into this direction.

The agents do cooperate in the aforementioned systems, by definition. But the occurrence of the opposite kind of interaction, competition, seems to be highly variable. It would be an interesting subject of research to review the relationship between cooperation and competition in these diverse systems. In plants, competition between the agents is ubiquitous. The nearby branches of roots compete for water and nutrients in the soil even within the same plant. Branches of the shoot system may shade each other, in competition for light. Competition between the agents for limited resources (or simply limited space) may be viewed in some systems as a factor that always diminishes the efficiency of the group. This is certainly not the case in plants. Competition can rather be considered as an important tool in their adaptive behaviour. For example, it enables the plant to support the more promising directions of growth, and to abandon the less promising ones ([4,10,11,19,33,57,94] and figure 3*b*).

The subject of the present issue of the *Philosophical Transactions of the Royal Society B* is the ‘solid’ versus ‘liquid’ nature of networks. I think that plants represent an interesting ‘hybrid’ between ‘solid’ and ‘liquid’. They are solid in the sense that the agents and links have fixed positions in space.^5^ The fact that the death of a link is irreversible also causes some rigidity in the behaviour of the system. But, despite of these limitations, the system is quite flexible. The agents are born and die unceasingly. The death of links can be decoupled from the death of agents. Flexibility is enhanced by the functional variability of agents and links, which also includes an opportunity for local specialization. In summary, plants show many interesting features in terms of gathering and processing information. Although they do not have any brain, their body itself is network-like, which enables complex kinds of adaptive behaviour.

Network theory has been applied on various levels of biological organization from molecules through cells and organs [119,120] up to ecological networks encompassing multiple species [121–123]. So, both the infra- and supraindividual levels have been studied. In modular organisms, however, there is a remarkable intermediate level, from the modules to the individual (genet), which has not been investigated from the view of network theory. To my knowledge, the present paper is the first systematic review of the topic in plants; and other modular organisms (corals, sponges, etc.) have not been reviewed. I think that the study of the networks of modules is crucial for obtaining a complete view biological organization. Within plants, it would be an exciting subject for future research to incorporate indirect interactions between the modules, mediated by mycorrhizae [113] or other organisms. It would be an important step further, from modular organisms to multi-species ecological networks.

## Footnotes

1 A commonly used synonym is ‘node’. To avoid any confusion, I use ‘nodus’ for the plant morphological term, and ‘node’ for the network theoretical one. It is interesting to observe how many technical terms are shared between botany and network theory/graph theory (e.g., tree, root, leaf, forest). This follows from the fundamental similarities between plants and some kinds of networks.

2 In physics and computer science ‘evolution’ is often used as a synonym for ‘change’. In biology, this is a more specific term, usually referring to a Darwinian process (or specified if meant otherwise). In this paper, I use the word in the biological sense.

3 Figure 1 depicts examples in which the offspring ramets originate from the shoot. They can originate from the root as well [44], for example, as in *Robinia pseudo-acacia*.

4 This simplification cannot be applied ubiquitously, for all clonal plant species. The stolons or rhizomes can have other roles than spacing and being transport channels. For example, the stolons in some species can significantly contribute to the total photosynthesis of the clone (e.g. in *Trifolium repens* [42]); and the rhizomes may considerably influence the resource economy of the plant by storing resources (e.g. in *Podophyllum peltatum* [39]). These species cannot be fitted into the Procrustean bed of this simple model; a more complex model is needed, for example, one in which the stolons or rhizomes are also represented as active agents.

5 Although fixing the position may take some time. For example, in the newly produced ramets of clonal plants, rooting may require days or even weeks [118]. For the sake of completeness, it should be noted that not all vascular plants are rooted (e.g. some aquatic species float in the water).

## Data accessibility

This article has no additional data.
